# The ubiquitin ligase Cullin5^SOCS2^ regulates NDR1/STK38 stability and NF-κB transactivation

**DOI:** 10.1038/srep42800

**Published:** 2017-02-20

**Authors:** Indranil Paul, Tanveer S. Batth, Diego Iglesias-Gato, Amna Al-Araimi, Ibrahim Al-Haddabi, Amira Alkharusi, Gunnar Norstedt, Jesper V. Olsen, Fahad Zadjali, Amilcar Flores-Morales

**Affiliations:** 1 Novo Nordisk Foundation Center for Protein Research, Department of Health and Medical Sciences, University of Copenhagen, 2200 Copenhagen N, Denmark; 2 Department of Drug Design and Pharmacology, Faculty of Health and Medical Sciences, University of Copenhagen, c/o the Danish Cancer Society, Strandboulevarden 49, DK-2100 Copenhagen Ø, Denmark; 3 College of Medicine and Health Sciences, Sultan Qaboos University, P.O. box 35, P.C 123, Muscat, Oman; 4 Department of Molecular Medicine, Karolinska Institutet, Stockholm, Sweden

## Abstract

SOCS2 is a pleiotropic E3 ligase. Its deficiency is associated with gigantism and organismal lethality upon inflammatory challenge. However, mechanistic understanding of SOCS2 function is dismal due to our unawareness of its protein substrates. We performed a mass spectrometry based proteomic profiling upon SOCS2 depletion and yield quantitative data for ~4200 proteins. Through this screen we identify a novel target of SOCS2, the serine-threonine kinase NDR1. Over-expression of SOCS2 accelerates turnover, while its knockdown stabilizes, endogenous NDR1 protein. SOCS2 interacts with NDR1 and promotes its degradation through K48-linked ubiquitination. Functionally, over-expression of SOCS2 antagonizes NDR1-induced TNFα-stimulated NF-κB activity. Conversely, depletion of NDR1 rescues the effect of SOCS2-deficiency on TNFα-induced NF-κB transactivation. Using a SOCS2^−/−^ mice model of colitis we show that SOCS2-deficiency is pro-inflammatory and negatively correlates with NDR1 and nuclear p65 levels. Lastly, we provide evidence to suggest that NDR1 acts as an oncogene in prostate cancer. To the best of our knowledge, this is the first report of an identified E3 ligase for NDR1. These results might explain how SOCS2-deficiency leads to hyper-activation of NF-κB and downstream pathological implications and posits that SOCS2 induced degradation of NDR1 may act as a switch in restricting TNFα-NF-κB pathway.

The suppressor of cytokine signaling 2 (SOCS2) is one of the substrate recognition modules of Cullin5/Rbx2 ubiquitin ligases. Classically, SOCS2 has been well-studied for its regulatory role on growth hormone (GH) signaling^1^,^2^. However, subsequent studies have found that this E3 ligase is an important regulator of inflammation. SOCS2 function is vital for maintaining immune homeostasis and its defects have been implicated in sepsis related mortality in mice models due to an exacerbated inflammatory response^3^,^4^. NF-κB signaling is centrally important to inflammatory processes^5^ and consequently, functional interactions between SOCS2 and NF-κB signaling have been studied^6^,^7^. We have previously described an inhibitory role of SOCS2 on NF-κB activation in macrophages and a recent study describe a similar finding in brain astrocytes^8^,^9^.

At the molecular level our understanding of SOCS2 function is limited. This is, in part, due to the pleiotropic nature of SOCS2^10^,^11^,^12^; but mostly due to our unawareness about its physiological protein substrates. Apart from GH receptor, substrates identified for SOCS2 till date, with relevance to inflammation, includes SOCS3^13^ and p-Pyk2^14^. SOCS2 destabilized SOCS3 and enhanced STAT signaling in response to IL-2/3 in T cells. Similarly, SOCS2 augmented IL-15 induced NK cell priming by degrading phospho-(Y402)-Pyk2. The status of NF-κB signaling were not evaluated in these studies. Clearly, the mechanisms of action of SOCS2 with respect to NF-κB signaling needs further exploration.

In order to identify and characterize bona-fide targets of SOCS2 we utilized mass spectrometry to quantify protein levels for thousands of proteins in cells depleted of SOCS2. This investigation lead to the identification of a number of proteins that could mediate the interplay between SOCS2 and NF-κB. In this investigation, the relationship between SOCS2 and NDR1 and its consequence for NF-κB activation is explored in detail.

## Results

### Quantitative proteomic screen of SOCS2 depleted cells identifies novel putative targets

**Figure 1:**
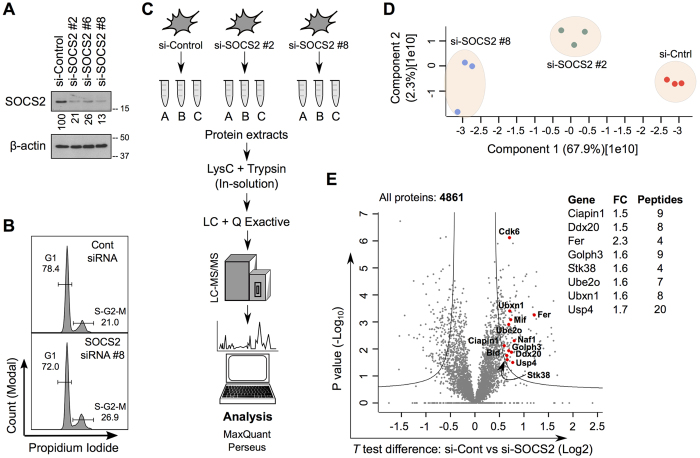
Global identification of proteins upregulated by SOCS2 depletion. (**A**) MEFs were transfected with either a non-targeting (NT) siRNA or three different siRNAs against SOCS2. Cell lysates were prepared 36 hrs after transfection and immunoblotted (IB) with the indicated antibodies. Numbers indicate % remaining protein, SOCS2/β-actin. (**B**) FACS analysis of MEFs transfected with si-SOCS2 #8. (**C**) Schematics of the proteomic workflow. (**D**) Principal component analysis of si-Control, si-SOCS2 #2 and si-SOCS2 #8 samples using LFQ intensities by the Perseus platform (n = 3). (**E**) Volcano plot of distribution of proteins identified in this study. Upregulated proteins (P-value < 0.05 and Fold change >1.5) reported in the literature to have a direct functional link with NF-κB are annotated. Right panel enlists genes corresponding to the annotated proteins which regulates NF-κB activity. “Fold change” and “unique peptides” are indicated.

We began our study by performing an unbiased proteomic screen to identify potential substrates of SOCS2. Physiological substrates of SOCS2 that are degraded in its presence are expected to accumulate when SOCS2 is depleted. We used an RNAi knock-down approach to avoid secondary effects of long-term SOCS2 depletion. Expression of SOCS2 was quantitated using immunoblotting (IB) (Fig. 1A). The overall effect on cell proliferation was measured by FACS (Fig. 1B), showing minor effects in the cell cycle upon SOCS2 depletion in mouse embryonic fibroblasts (MEFs). We characterized the proteome of SOCS2 wild-type (SOCS2^WT^) and knock-down (SOCS2^KD^) MEFs using nanoLC-MS/MS (nanoscale liquid chromatography coupled to tandem mass spectrometry) based proteomics (Fig. 1C). Three independent experiments were carried out using two different siRNAs against SOCS2, resulting in biological triplicates of essentially two biological groups. MS data of the biological triplicates from both groups were combined and analyzed by using a combination of MaxQuant and Perseus (see Methods). Relative label-free quantification (LFQ) was fairly reproducible between the triplicates, while some variation was observed across the different groups as depicted by principle component analysis (Fig. 1D). Overall, using this approach we detected ~5000 proteins and document quantitative data for >4200 proteins upon SOCS2 depletion (Fig. 1E). Expression of most proteins (94.6%) were unaltered and only a very small subset of proteins (5.4%), which satisfied the criteria of a fold-change (±) of ≥1.5 and P-value < 0.05, showed differences between SOCS2^WT^ and SOCS2^KD^ MEFs (Table 1 and Table S1). No expression of SOCS2 or its previously identified target, the GHR (growth hormone receptor), was detected most likely due to their low abundances^15^.

### Both endogenous and exogenously expressed NDR1 is destabilized by SOCS2

**Figure 2:**
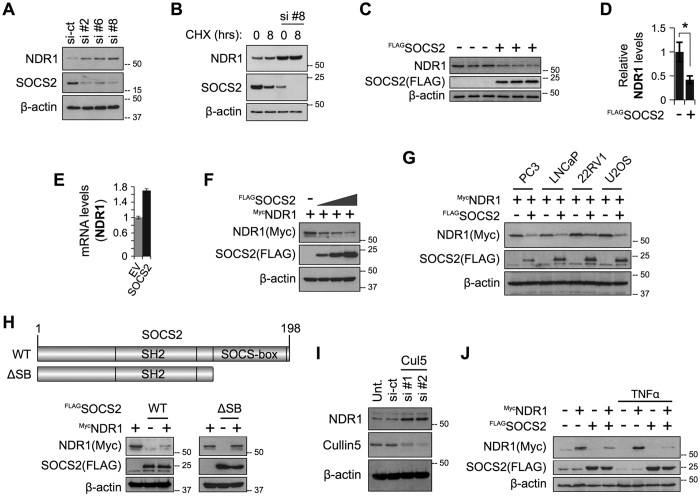
SOCS2 destabilizes NDR1. (**A**) Blots prepared in Fig. 1A were re-probed for NDR1. (**B**) MEFs were transfected with either a NT siRNA or si-SOCS2 #8 and 24 hrs later treated with cycloheximide for additional 8 hours. Lysates were propped by IB. (**C**) MEFs were transfected with FLAG-SOCS2 plasmid. Lysates from three independent experiments were probed by IB. (**D**) Pixel densitometry values obtained using ImageJ and normalized against β-actin are shown as a bar graph representing mean ± SEM, *P value < 0.05. (**E**) QPCR analysis of NDR1 gene using total RNAs extracted from MEFs transfected with FLAG-SOCS2 or an empty vector (EV). 18S rRNA was used as internal control. (**F**) MEFs were transfected with a constant amount of a plasmid encoding Myc-NDR1 and an increasing amount of FLAG-SOCS2, while keeping the total amount of DNA equal using EV. IB was done using indicated antibodies. (**G**) Plasmid for Myc-NDR1 were co-transfected with either EV or FLAG-SOCS2 in a panel of different cell lines and lysates were probed by IB. (**H**) MEFs were transfected with Myc-NDR1 in combination with either FLAG-SOCS2 or FLAG-SOCS2-ΔSB plasmids as shown. Lysates were probed by IB. (**I**) RNAi mediated depletion of Cullin5 in MEFs and IB for NDR1. (**J**) Plasmid for Myc-NDR1 were co-transfected with either EV or FLAG-SOCS2 and treated with TNFα (10 ng/ml for 1 hour) as shown. Lysates were probed by IB. Full-length blots of 2C (SOCS2) and 2C (β-actin) blots are presented in Supplementary Figure S4.

Bioinformatics analysis and text mining of significantly upregulated proteins in our dataset identified a handful of proteins previously associated with the regulation of NF-κB activity (See Fig. 1E). Literature review indicated that FER, GOLPH3 and STK38 upregulates NF-κB activity^16^,^17^,^18^. Given the recently published link between NDR1(STK38) and TNFα-induced NF-κB activation, we focussed our attention on NDR1. To investigate the possibility that SOCS2 regulates NDR1, we first used immunoblotting (IB) to verify that depletion of SOCS2 by siRNAs indeed increased the cellular levels of endogenous NDR1 protein under resting conditions (Fig. 2A) and when *de novo* protein synthesis has been blocked using cycloheximide (Fig. 2B). Over-expressed ^FLAG^SOCS2 could decrease endogenous NDR1 protein but not mRNA (Fig. 2C–E). Furthermore, ^FLAG^SOCS2 could destabilize exogenously expressed ^Myc^NDR1 in MEFs in a dose-dependent manner (Fig. 2F) and in a panel of different cell lines (Fig. 2G). We also observed that ^FLAG^SOCS2^WT^ but not ^FLAG^SOCS2^ΔSB^ (deletion of SOCS box which is required for interaction with ElonginB/C and Cullin5 complex) mutants could decrease levels of endogenous NDR1 protein (Fig. 2H), suggesting that the SOCS-box domain is required for SOCS2 to regulate NDR1 abundance, likely mediated through the Cullin5 E3 ligase. Consistently, depletion of Cullin5 increased NDR1 protein levels (Fig. 2I). Over-expressed ^Myc^NDR1 did not have any detectable effects on the total protein levels of exogenous ^FLAG^SOCS2 in MEFs (Fig. 2H). We also observed that the destabilizing effect of ^FLAG^SOCS2 on ^Myc^NDR1 is further enhanced after treatment with TNFα (Fig. 2J). It is known that the interaction of SOCS2 with its target is mediated through phosphorylated tyrosine (Y) residue(s) on the substrate. Substitution of several of the Y residues on NDR1 with high probability of phosphorylation (source: PhosphoSitePlus database), could not abolish the ability of SOCS2 to degrade NDR1 (Figure S1), suggesting requirement of either multiple phospho-Y sites or Y residues other than the mutated ones. Collectively, these data demonstrate that NDR1 protein is regulated by SOCS2.

### SOCS2 interacts with NDR1 leading to its polyubiquitination

**Figure 3:**
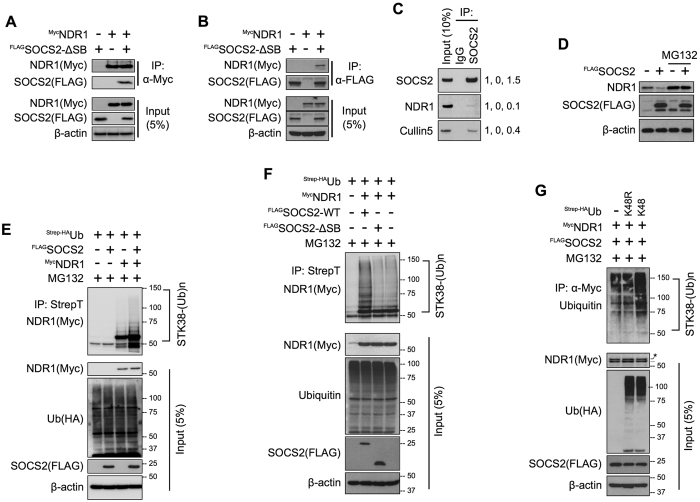
SOCS2 interacts with NDR1 and promotes its ubiquitin-mediated degradation. (**A,B**) FLAG-SOCS2-ΔSB and Myc-NDR1 were expressed in MEFs either alone or together. Lysates were immunoprecipitated with anti-Myc (9E10) antibody (**A**) or anti-FLAG M2 antibody (**B**) and subjected to SDS–PAGE followed by IB. (**C**) HEK293T cells grown to ~80% confluency were harvested. Lysates (2 mg) were subjected to IP with anti-IgG or anti-SOCS2 antibody followed by IB with the indicated antibodies. Values on the right are densitometric quantifications with the corresponding “Input” band taken as 1. (**D**) MEFs were transfected with FLAG-SOCS2 plasmid as shown. Cells were treated with 10 μM MG132 or an equal volume of DMSO for 16 hrs before harvesting. Lysates were prepared and IB was done as previously. (**E,F**) Plasmids for HA-Strep-Ubiquitin and Myc-NDR1 were co-expressed in MEF cells with FLAG-SOCS2 (**E**) or FLAG-SOCS2 and FLAG-SOCS2-ΔSB (**F**) as shown. Cells were treated with 10 μM MG132 or an equal volume of DMSO for 16 hrs before harvesting. Lysates were prepared from 36 hrs post-transfected cells and subjected to pull-down using StrepTactin resin followed by IB. (**G**) Plasmids for HA-Strep-Ubiquitin-K48 or K48R and Myc-NDR1 were co-expressed in HEK293T cells with FLAG-SOCS2 as shown. Cells were treated with 10 μM MG132 or an equal volume of DMSO for 16 hrs before harvesting. Lysates were prepared from 36 hrs post-transfected cells and subjected to pull-down using anti-Myc (9E10) antibody followed by IB. Lysates were probed by IB. Full-length blot of 3D (NDR1) are presented in Supplementary Figure S4.

Consistent with a role of SOCS2 in regulating NDR1 stability, over-expressed ^Myc^NDR1 was detected in exogenously expressed ^FLAG^SOCS2^ΔSB^ pull-down complexes and *vice versa* in HEK293T cells (Fig. 3A,B). We used a SOCS-box deleted form of SOCS2 for the pull-down assays to prevent SOCS2-induced degradation of NDR1 and to avoid Cullin5-interacting proteins in the immunoprecipitation. This interaction was confirmed in MEFs under endogenous conditions (Fig. 3C). We previously observed that ^FLAG^SOCS2 over-expression could decrease levels of endogenous NDR1. This effect of SOCS2 on NDR1 is reversed by MG132 (26 S proteasome inhibitor) treatment (Fig. 3D), indicating that SOCS2 triggered reduction in NDR1 requires activity of the 26 S proteasome. Indeed, we observed a quantitative increase of high molecular weight polyubiquitinated forms of ^Myc^NDR1 upon ^FLAG^SOCS2^WT^ over-expression (Fig. 3E) but not ^FLAG^SOCS2^ΔSB^ over-expression (Fig. 3F) and these polyubiquitination was through K48-linkage (Fig. 3G). On the basis of above data, we propose that SOCS2 destabilizes NDR1 by promoting ubiquitination and its subsequent degradation by the 26 S proteasome.

### SOCS2 deficiency stimulates NF-κB activation in response to TNFα

**Figure 4:**
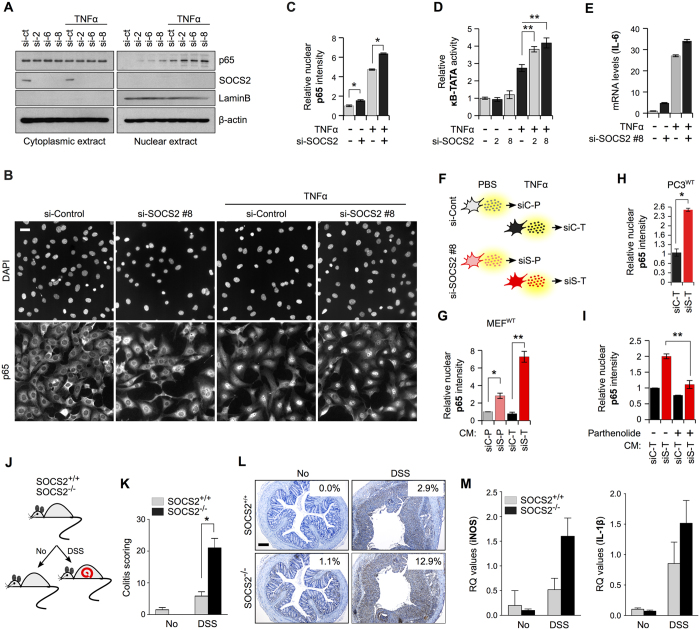
SOCS2 inhibits TNFα-induced NF-κB signaling. (**A**) MEFs were transfected with either NT or 3 different SOCS2 siRNAs. After 36 hours cells were treated with TNFα (10 ng/ml) for 45 mins and cytoplasmic and nuclear fractions were probed with the indicated antibodies. (**B**) MEFs were transfected with NT or SOCS2 siRNA as shown. After 36 hrs cells were treated with TNFα (10 ng/ml) for 45 mins or left untreated before being processed for immunofluorescence microscopy against p65 using HCQI. Scale bar represents 100 μm. (**C**) Automated unbiased quantitation of nuclear p65 staining intensity was done using the ScanR software. Mean fluorescence intensity of p65 per nucleus for each ‘plate’ were determined. Data is representative of two independent experiments and depicts relative fold change ± standard error. (**D**) MEFs grown in 60 mm dishes were transfected with NT or SOCS2 siRNA along with pGL4.10-NF-κB (100 ng) and pGL4.74-(hRluc/TK (10 ng) as shown. After 36 hrs cells were either treated with TNFα (10 ng/ml for 6 hrs) or left untreated. Bar graph represents normalized luciferase readings against Renilla reporter activity for three independent experiments. (**E**) Q–PCR analysis of NF-κB target gene IL-6 was performed using total RNAs extracted from MEFs transfected with NT or SOCS2 siRNAs and treated with TNFα (10 ng/ml for 1 hour). 18S rRNA was used as internal control. (**F**) Schematics of the treatments of MEFs for preparing conditioned media (CM). (**G**) Serum-starved MEFs were exposed to CM prepared above for 1 hour before being processed as in (**B**) and quantitated as in (**C**). (**H**) Serum starved (16 hrs) PC3 cells were treated as in (**G**) with the indicated CM and quantified as in (**C**). (**I**) Serum-starved MEFs were exposed to CM, prepared as in (**F**) with the exception that parthenolide was added to the cells together with TNFα as indicated, for 1 hour before being processed as in (**B**) and quantitated as in (**C**). (**J**) Schematics showing the DSS treatment for inducing ulcerative colitis in mice. (**K**) Representative images of colon tissues from SOCS2^+/+^ and SOCS2^−/−^ mice exposed to DSS as indicated in (**J**) and probed for p65 using immunohistochemistry. (**L**) Histology scoring for colitis based on multiple parameters (see Methods section). White boxes on each image represent a staining index quantified by ImmunoRatio web application using default parameters^47^. Scale bars represent 200 μm. (**M**) Q–PCR analysis of the indicated genes in total RNA isolated from colonic tissues of SOCS2^+/+^ and SOCS2^−/−^ mice exposed to DSS as indicated in (**D**). For all Figures, *P value < 0.05 and **P value < 0.005.

Because NDR1 potentiates NF-κB signaling in the presence of TNFα ligand^18^, we wished to evaluate the effect of SOCS2 on this aspect of NDR1 function due to its potential implications for clarifying the mechanisms behind SOCS2 anti-inflammatory actions, as mentioned above. However, before delving on to the functional implications of SOCS2 perturbations on NDR1-potentiated NF-κB signaling, we first asked what is the role of SOCS2 in TNFα-induced NF-κB activation in MEFs under our experimental conditions. IB analysis of cytoplasmic and nuclear extracts showed that MEFs depleted of SOCS2 (SOCS2^KD^) had significantly higher nuclear p65 levels, as compared to wild-type (SOCS2^WT^) cells, after stimulation with TNFα (Fig. 4A). We obtained similar results using high-content quantitative imaging (HCQI) (Figure S2A, B and Fig. 4B,C) which allowed detection of nuclear p65 quantitatively *in situ*^19^,^20^. Consistently, SOCS2^KD^ cells were hyper-responsive to TNFα ligand with respect to NF-κB activity as determined by luciferase reporter assay (Fig. 4D) and changes in mRNA levels of the NF-κB target gene IL-6 (Figure S2C and Fig. 4E). We also observed that SOCS2 depletion resulted in changes in proteins secreted by TNFα-stimulated MEFs that promoted NF-κB transactivation in wild-type cells (i.e., in a paracrine manner) (Fig. 4F–I). We contextualize our *in vitro* data in an *in vivo* setting by using the murine DSS (dextran sodium sulphate) model of ulcerative colitis (UC)^21^ where TNFα is a major driving factor^22^. DSS-administered induction of UC has not been previously reported in SOCS2 knock-out (SOCS2^−/−^) mice. We found that in comparison to wild type mice, colonic tissue from SOCS2^−/−^ mice showed exacerbated inflammation upon DSS treatment (Fig. 4J,K). Consistently, we observed much stronger nuclear staining for p65 in SOCS2^−/−^, as compared to SOCS2^WT^, mice after DSS administration (Fig. 4L) which was reflected in higher transcript levels of known NF-κB target genes, iNOS and IL-1β (Fig. 4M). As a whole, our observations indicate that SOCS2 depletion facilitates NF-κB signaling.

### NDR1-mediated TNFα-induced NF-κB transactivation is inhibited by SOCS2

**Figure 5:**
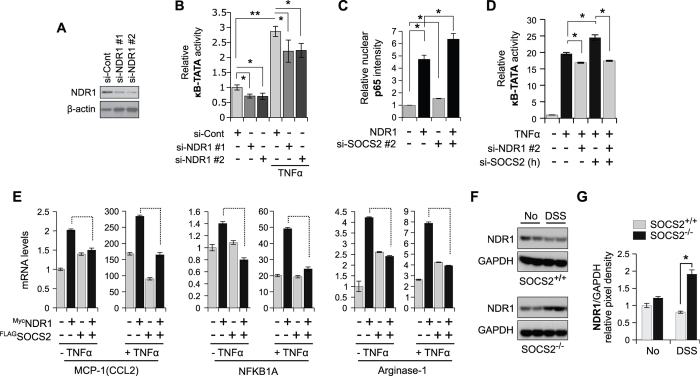
SOCS2 inhibits TNFα-NDR1-NF-κB axis. (**A**) Efficacy testing of siRNAs in MEFs against NDR1 using IB. (**B**) MEFs were transfected with NT siRNA or NDR1 siRNAs. After 36 hrs cells were treated with TNFα (10 ng/ml) for 1 hour and luciferase assay was performed as in Fig. 4D. (**C**) MEFs were cotransfected with a plasmid for Myc-NDR1 and SOCS2 siRNA as shown. After 36 hrs cells were treated with TNFα (10 ng/ml) for 1 hour before being fixed and processed for HCQI. Nuclear p65 intensity values were quantified and plotted as in Fig. 1C. (**D**) HEK293T cells were transfected with NT or SOCS2 siRNA (human) as shown and luciferase assay was performed as in Fig. 4D. (**E**) Quantitative RT-PCR analysis of NF-κB target genes was performed using total RNAs extracted from MEFs transfected with FLAG-SOCS2 & Myc-NDR1 and treated with TNFα (10 ng/ml for 1 hour) or left untreated as shown. (**F**) Evaluation of endogenous protein levels of NDR1 using IB in extracts prepared from colonic tissues of SOCS2^+/+^ and SOCS2^−/−^ mice, pre- and post-DSS insult as shown. (**G**) Pixel densitometry values of NDR1 bands obtained using ImageJ and normalized against GAPDH are shown as a bar graph. SE values are averages of densitometry readings from two representative mice. For all Figures, *P value < 0.05 and **P value < 0.005.

Having observed that SOCS2 depletion promotes several aspects of TNFα-induced NF-κB signaling in MEFs, we next asked how does this correlate to NDR1. In MEFs, transient over-expression of NDR1 amplifies TNFα-induced NF-κB signaling (Figure S3A–C). Consistently, depletion of NDR1 causes reduction of NF-κB dependent luciferase activity in both presence and absence of external TNFα stimulation, although the effect is more pronounced in presence of TNFα (Fig. 5A,B). Using HCQI we show that over-expression of NDR1 and down-regulation of SOCS2 had a combined additive effect on TNFα-induced nuclear translocation of p65 (Fig. 5C). Further, depletion of SOCS2 increased luciferase readout over TNFα stimulation alone, while knock-down of either NDR1 or double knock-down of both NDR1 and SOCS2 reduced NF-κB luciferase reporter activity below that of TNFα treatment (Fig. 5D) suggesting NDR1 to be downstream of SOCS2. Conversely, SOCS2 over-expression should diminish the strengthened transactivation potential of NF-κB in response to TNFα. To test this, we measured mRNA levels of well-known NF-κB target genes (MCP-1, NFKB1A and Arginase-1) using quantitative real-time PCR (qPCR) analysis upon NDR1 and SOCS2 overexpression either alone or in combination in MEFs (Fig. 5E). We observed that overexpression of SOCS2 alone stimulated the transcription of these genes. This is not unexpected given these genes may not be exclusively regulated by NF-κB under resting conditions and apparently depend on unidentified factors that are influenced by SOCS2. Nevertheless, NDR1 had an augmenting effect on the transcription of all the genes tested, and importantly, in all instances SOCS2 truncated this effect (Fig. 5E). We also provide a correlative support to our *in vitro* observations by showing that inflamed SOCS2^−/−^ colonic tissues isolated from DSS-treated mice had elevated total levels of NDR1 protein as compared to similarly treated wild-type mice (Fig. 5F,G).

As a whole, our observations potentially point towards an explanation for the effects of SOCS2 on NF-κB activity and the hyper-inflammatory phenotype of SOCS2 deficient animals.

### NDR1 amplifies the aggressive behaviour of PC3 cells

**Figure 6:**
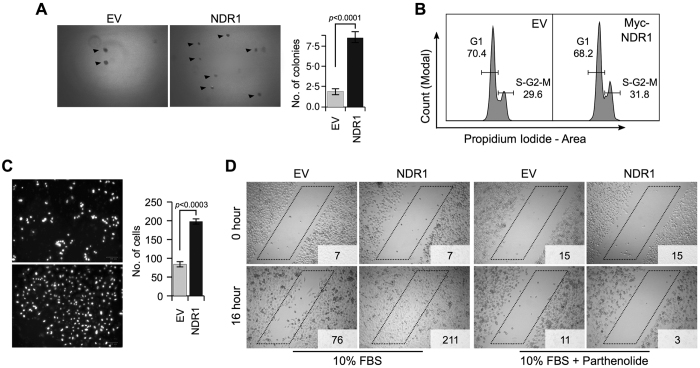
NDR1 acts as an oncogene in PC3 cells. (**A**) Representative images of soft-agar colony formation assay in PC3 cells transfected with EV or NDR1 plasmid. Bar graph displays data from three independent experiments and represents mean ± standard error. (**B**) Cell cycle profile from FACS analysis of cells transfected as in (**A**). (**C**) Representative images of matrigel invasion assay of PC3 cells transfected as in (**A**). Bar graph displays data from three independent experiments and represents mean ± standard error. (**D**) Representative images of “wound-healing” assay in PC3 cells transfected as in (**A**) and treated with NF-κB inhibitor parthenolide as shown. Numbers in insets represents cell count within the “box”.

We previously observed that SOCS2 acts as a tumour suppressor in advanced castration-resistant prostate cancer. Bone metastases clinical samples of human patients exhibit reduced levels of SOCS2 in comparison with localized or hormone naive untreated adenocarcinomas^23^. A majority of these advanced tumors are known to have an elevated dependency on NF-κB signaling for their survival^24^. Since SOCS2 deficiency could lead to an elevated NDR1 levels, we wanted to see how an “over-expressing” NDR1 protein itself would impinge on the tumorigenic properties of cancer cells. As per our knowledge this has not been tested before. For this we selected PC3 prostate cancer cells, partly because of their “addiction” to NF-κB signaling^25^,^26^. To determine whether NDR1 over-expression alters the anchorage-independent growth of PC3 cells we performed soft-agar colony-formation assay and observed a marked difference in the number of colony-forming units. NDR1-overexpression in PC3 cells (PC3-NDR1) potentiated sphere-forming capacity by ~2.9-fold as compared to cells transfected with empty vector (PC3-EV) (Fig. 6A). However, PC3-NDR1 cells did not appear to have a significantly altered cell cycle profile in comparison to PC3-EV cells as measured using FACS analysis (Fig. 6B). PC3-NDR1 cells also had relatively higher invasive potential through Matrigel chamber (Fig. 6C). We next performed 2-dimensional wound-healing assay to test the migratory behaviour of these cells and found that PC3-NDR1 cells repopulated the cell-free clearing produced by the scratch faster than PC3-EV cells, indicating an increased migration rate. Importantly, inclusion of the NF-κB inhibitor parthenolide abrogated wound-healing capacity of both PC3-EV and PC3-NDR1 cells (Fig. 6D) highlighting the crucial role played by NF-κB in these cells. Taken together, these data demonstrate that NDR1 might act as a driver of oncogenic growth in NF-κB addicted tumors, although further research is needed to fully justify this claim.

## Discussion

As mentioned above, the intracellular mechanisms by which anti-inflammatory actions are exerted by SOCS2 are poorly understood. This problem is related with our scarce knowledge of its cellular targets. To shed light on this aspect, we performed an unbiased nanoLC-MS/MS based quantification of cellular proteins in MEFs after transient knock-down of SOCS2 and enlisted 136 upregulated proteins. Of note, based on this experimental design we cannot rule out the contribution of secondary effects to changes in protein concentration, and the numbers of direct targets of SOCS2 are likely to be lower. Thus, additional research is needed to establish whether and how proteins identified in this analysis as putative SOCS2 targets are indeed ubiquitinated by SOCS2 and how they contribute to its cellular functions, including inflammation. Nonetheless, as it stands, this is the first report of a global quantitative proteomics effort specifically directed towards identification of SOCS2 regulated proteins.

Our proteomic data revealed NDR1 as a potential target for SOCS2 ubiquitin ligase activity. NDR1 (or STK38) is a serine-threonine kinase. It belongs to the LATS/NDR family and controls many critical processes including centrosome duplication^27^, apoptosis and proliferation^28^, chromosome alignment^29^. It regulates signaling pathways such as MEKK1/2^30^, TGFβ^31^, Hippo^32^ and TLR9^33^. Recently, NDR1 was shown to potentiate NF-κB signaling in the presence of TNFα ligand in HEK293T and HeLa cells^18^. Evidences indicate NDR1 protein is highly regulated through phosphorylation^32^ and the ubiquitin proteasome system^31^. Several kinases, including MST1^34^ and GSK3β^35^ are known to regulated NDR1 activity under various contexts. However, no physiological E3 ligase had yet been reported for NDR1.

Using immunoprecipitation, we show that SOCS2 and NDR1 exist in the same complex. NDR1 was found to be a substrate of wild-type SOCS2 but not a SOCS-box truncated form i.e., SOCS2-ΔSB. SOCS2 acts as the substrate recognition module of Cullin5/Rbx2 E3 ligase “holoenzyme” and depends on its C-terminus SOCS box to interact with the complex, suggesting the involvement of Cullin5 E3 ligase. Consistently, knock-down of Cullin5 using RNAi stabilized NDR1 protein. Overexpression of SOCS2 increases polyubiquitination of NDR1 subsequently targeting it to 26S proteasomes as indicated by an increased ubiquitin-NDR1 conjugates in presence of MG132. We also provide data to support that SOCS2 ubiquitination of NDR1 is mediated mainly by K48-Ub-specific linkages that are mostly associated with proteolysis. Conversely, our data showing stabilization of NDR1 upon SOCS2 depletion indicates a constitutive ligase-substrate relation between them.

Considering some of the latest evidences, it appears that SOCS2 probably orchestrates multiple regulatory nodes in the inflammatory pathway^8^,^36^,^37^,^38^. We observed that over-expression of NDR1 alone had a two-fold enhancing effect on NF-κB transactivation, as judged by qPCR of well-known NF-κB target genes. Not surprisingly, the overall impact was much stronger when the cells were induced with TNFα but the fold change was still ~2.0 fold. Therefore, our report agrees with Shi *et al*., that NDR1 potentiates TNFα induced NF-κB signaling, however, the mechanism behind this effect is yet to be uncovered. Here we report SOCS2 induced destabilization of NDR1. SOCS2 can constitutively degrade NDR1 without an activating stimulus (other than provided by standard cell culture conditions), although interestingly, a TNFα stimulus seem to make NDR1 a better target of SOCS2. Based on our proteomics observations, we believe that NDR1 protein is relatively more abundant than SOCS2 because we repeatedly detected NDR1 by mass spectrometry analysis but not SOCS2. This might mean that manipulating SOCS2 levels may serve a switch for controlling TNFα-NF-κB signaling mediated through NDR1.

We offered evidence that SOCS2 regulation of NDR1 may be relevant for inflammation and carcinogenesis. Our observations showing elevated tumorigenic properties of PC3 cells in response to NDR1 over-expression raises interesting questions and begs further research into the carcinogenic role of NDR1. Given the role of NF-κB signaling in multiple human malignancies, our study raises the potential for SOCS2-NDR1-NF-κB axis as a target for drug discovery in inflammation and cancer biology.

**Figure 7:**
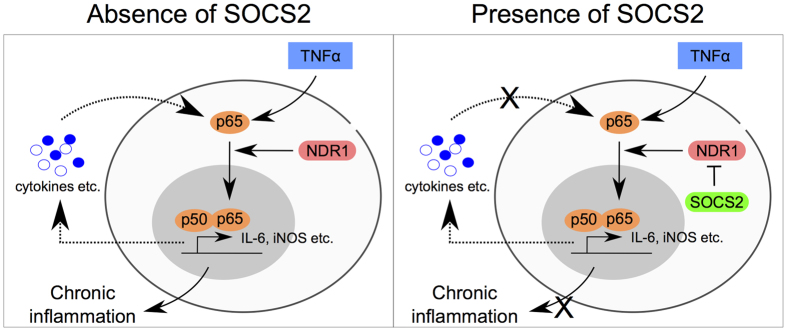
A simplified model of SOCS2 mediated regulation of TNFα - NF-κB signaling through NDR1 ubiquitination and degradation.

Overall, the data presented in this study (1) provides evidence for an inhibitory role of SOCS2 in TNFα induced NF-κB activation, (2) identifies NDR1 as a novel substrate of SOCS2, and (3) demonstrates that SOCS2 and TNFα induced NF-κB signaling are linked through NDR1. Through this study, we provide a mechanistic explanation for the well-acknowledged hyper-inflammatory phenotype of SOCS2-deficient animals (Fig. 7).

## Materials and Methods

### Cell Culture, transfection and drug treatments

Cells were cultured using standard protocols. Transfections of DNA constructs and siRNAs were performed using Neon^®^ Transfection System (Thermo Fisher Scientific) according to the manufacturer’s instructions. The following drugs/inhibitors were used: MG132, cycloheximide (Sigma-Aldrich), mouse and human recombinant TNFα (Invitrogen). Concentrations used and incubation times are given in Figure legends.

### Expression plasmids and siRNA

Human SOCS2 gene (NM_003877) was PCR amplified from total HEK293T cDNA. Full-length SOCS2 and SOCS2^ΔSB^ deletion mutant (amino acids 1–145) were cloned into pcDNA 4/TO vector with an N-terminal FLAG epitope to generate ^FLAG^SOCS2^WT^ and ^FLAG^SOCS2^ΔSB^. pcDNA4/TO-HA-Strep-ubiquitin WT, K48-only and K48R were a kind gift from Niels Mailand (University of Copenhagen). Myc-tagged NDR1 was obtained from Addgene (Plasmid #37023, a kind gift from Yutaka Hata). Point mutations of Myc-NDR1 were created using the site-directed mutagenesis kit (Agilent Technologies) as per manufacturer’s instructions. All plasmids were verified by sequencing. Sequences of primers used can be provided upon request. All siRNAs were purchased from Qiagen and used at a final concentration of 80 nM. Sequences are given in Table S2.

### Preparation of conditioned medium

Cells were starved in serum-free media for 16 hours before being pulsed with TNFα (10 ng/ml) for 1 hour after which residual TNFα was washed twice using PBS. Serum-free media was again replenished and cells were inubated for another 4 hours. CM were collected, clarified by centrifugation and passed through sterile 0.22 μm sterile filter. CM were used freshly.

### Co-immunoprecipitation (co-IP) and immunoblot (IB) analyses

For co-IP experiments, cells were lysed on ice using IP buffer (50 mM HEPES, pH 7.2, 250 mM NaCl, 10% glycerol, 1% Nonidet P-40, 1.0 mM EDTA, 10 mM phenylmethanesulfonylfluoride (PMSF), 50 mM sodium fluoride, 0.2 mM sodium orthovanadate (Na_3_VO_4_), 25 mM β-glycerophosphate and protease inhibitor cocktail from Roche). After pre-clearing with Protein A Sepharose beads (Amersham Biosciences), 2 mg of total protein was subjected to immunoprecipitation as described previously^39^. For western blotting, cells were lysed on ice using RIPA buffer (50mM Tris pH 7.4, 150 mM NaCl, 0.1% SDS, 0.5% sodium deoxycholate, 1% Triton X 100, 10 mM phenylmethanesulfonylfluoride (PMSF), 50 mM sodium fluoride, 0.2 mM sodium orthovanadate (Na_3_VO_4_), 25 mM β-glycerophosphate and protease inhibitor cocktail from Roche). The following antibodies were used: *Santacruz Biotechnology* - GAPDH, Myc (9E10), NDR1(STK38), Ubiquitin; *Sigma* - β-actin, HA, FLAG(M2); *Abcam* – SOCS2; *Abnova* – NDR1; *Cell Signaling Technology* – SOCS2, p65, FLAG. Pixel densitometric measurements of immunoblots was done using the ImageJ software (https://fiji.sc/).

### Orbitrap Q-Exactive MS/MS and proteomics data analysis

Lysates were prepared as described previously^40^. From each sample, 100 μg of proteins were first digested with Lys-C (4 hours at 37 °C) followed by dilution of guanidine hydrochloride to <2 M with 50 mM ammonium bicarbonate prior to the addition sequencing grade Trypsin (overnight at 37 °C). Enzyme activity was quenched by acidification of the samples with trifluoroacetic acid (TFA) and the peptide mixture was desalted and concentrated on a C_18_ Stagetip before elution with 50% acetonitrile, 0.1% TFA. The eluate was concentrated in a SpeedVac at 60 °C and acidified with 5% ACN, 1% TFA before being analyzed using an EASY-nLC system (Proxeon) connected to a Q Exactive mass spectrometer (Thermo Fisher Scientific) through a nano-electrospray ion source. Peptides were separated in a 15-cm analytical column (75 μm inner diameter) in-house packed with 1.9 μm diameter reversed-phase C_18_ beads (ReproSil-Pur AQ, Dr. Maisch) with an increasing gradient from 5 to 30% buffer B (80% acetonitrile, 0.1% formic acid) in 120 minutes at a flow rate of 250 nl/min followed by an increase to 40% B in 15 minutes where it was briefly held prior to 80% B for 5 minutes followed by column re-equilibration at 5% B. The Q-Exactive was operated in data-dependent acquisition mode using the “sensitive scanning method”^41^. Raw MS files from the Q-Exactive were analyzed by MaxQuant 1.4.1.1 with the Andromeda search engine^42^. Tandem mass spectrometry spectra were searched against the decoy International Protein Index-Mus musculus database version 3.62 containing both forward and reverse protein sequences. The search included variable modifications of methionine oxidation and N-terminal acetylation, and fixed modification of cysteine carbamidomethylation. Peptides of minimum seven amino acids and maximum of two missed cleavages were allowed for the analysis. False discovery rate of 1% was used for the identification of peptides and proteins. Bioinformatics analysis of the data were mainly performed using the Perseus computational platform^43^ and text mining in PubMed using the search string: ((NF-κB[Title]) AND gene1[Title]) OR gene2[Title] etc. for all identified proteins with a P-value < 0.05 and Fold change >1.5.

### Luciferase assays

Luciferase activity of NF-κB reporter was determined luminometrically using the Dual-Glo luciferase assay system (Promega). MEFs (10^6^) were transiently transfected with pGL4.74-hRluc/TK (10 ng) and pGL4.10.NF-κB reporter (100 ng) vectors along with the indicated constructs. Luciferase activity of cell extracts in passive lysis buffer was determined luminometrically using the Varioskan multiplate reader (Thermo Fisher Scientific).

### Microscopy

Protocol has been described before^39^. For High-Content Quantitative Imaging (*HCQI*), photographs were captured on Olympus IX-81 motorized fluorescence microscope equipped with an Olympus UPLSAPO 20x/0.75 NA objective. Automated and unbiased image analysis was carried out with the ScanR acquisition software. For confocal fluorescence microscopy, images were captured on a Zeiss LSM 700 imaging system.

### Quantitative real-time PCR (qPCR)

Total RNA was extracted using the RNeasy kit (Qiagen). For each sample, 1 μg of RNA was converted to cDNA using iScript cDNA synthesis kit (Biorad), diluted 1:5 with water and subsequently used for qPCR analysis using Brilliant III ultra-fast SYBR^®^ green (Agilent Technologies) on a 7500 Fast Real-Time PCR system (Applied Biosystems). In all experiments, 18S rRNA served as the internal control (normalization) and calibrator controls were chosen appropriately. Sequences of the primers used are given in Table S3.

### Immunohistochemistry

Tissue sections were deparaffinized in xylene and rehydrated through graded ethanol. For antigen retrieval slides were steamed for 30 minutes in EnVision FLEX Target Retrieval Solution (pH 6.1) (Dako). Antibody incubations were done overnight at 4 °C in Shandon coverplates (Thermo Fisher Scientific). For detection, HiDef HRP polymer system (Sigma-Aldrich) was used followed by incubation with DAB + chromogen (DAKO) according to manufacturer’s instructions. Slides were counterstained with Mayers hematoxylin for 1 minute. All immunohistochemical procedures were undertaken in one batch. The antibody for p65 was from Cell Signaling Technology.

### Laboratory Animals and Experimental Colitis

Mice were housed under standard conditions [12:12-hour light-dark cycles, 22 ± 2 °C and about 60% humidity] with *ad libitum* feeding. Male SOCS2 knockout (SOCS2−/−) and SOCS2-wild type (SOCS2+/+) littermate mice between 12–16 weeks’ old were used and they were allowed to acclimate to these conditions for a week before the start of the experiment. Colonic inflammation was induced by oral administration of 3% (w/v) Dextran Sodium Sulfate (DSS; T&D consulting, Uppsala, Sweden) dissolved in drinking water for 5 days and then switched to normal drinking water until day 20. Mice were sacrificed after euthanasia by total bleeding and cervical dislocation after anesthesia by isoflurane. Entire colons of DSS treated and untreated mice were dissected and contents flushed with ice-cold saline. The distal third of the colon was dissected into equal pieces for histological analysis and RNA & protein extractions. Following H + E staining, specimens were evaluated for histological colitis scoring by two independent pathologist using light microscopy. The scoring of histological changes in the colon was done according to Cooper *et al*.^44^, which include the assessment of Crypt loss, infiltration of neutrophil, lymphocytes and goblet cell depletion^45^,^46^. Histological findings were given a score based on the severity of the colitis (Table S4). Animal handling and all experimental protocols were performed in accordance with the relevant guidelines and regulations approved by the “Animal Ethics Committee” of Sultan Qaboos University, Oman.

### Soft-agar colony formation assay

For soft-agar assays, 300 cells were suspended in RPMI containing 0.35% agar and layered on top of 1 ml of RPMI solidified with 1.0% agar in each 35-mm dish. After growing at 37 °C for 14 days, colonies were visualized and counted in three different microscopic fields.

### Wound healing assay

The migratory behavior of PC3 cells were evaluated using the scratch (wound healing) assay. Briefly, equal number of cells were seeded on 6-well plates and allowed to reach confluency. Several scratches were made using a 200 μl pipette tip spanning the well. Migration of cells was observed at premarked positions below the wells using an Olympus IX81 inverted microscope at 40X magnification.

### Matrigel invasion assay

Matrigel (60 μl at 1:4 dilutions in RPMI medium) was coated on the top of Transwell filters (6.5-mm diameter, 8.0-μm pore size, Costar, Corning, NY) for 4 h at 37 °C. Chemoattractant (10 ng/ml TNFα) was added to bottom chambers of transwells and cells (50,000) were plated on Matrigel in RPMI and allowed to migrate overnight at 37 °C through Matrigel. Migrated cells were fixed in 4% paraformaldehyde in PBS for 15 minutes and permeabilized with 0.2% Triton -X100 in PBS for 10 minutes. Cells migrated to the lower chamber were stained with 0.5 μg/ml DAPI in PBS. Fluorescence pictures were acquired using an Olympus IX81 inverted microscope at 40X magnification from three random fields and nuclei were counted using ImageJ.

### Statistical analysis

All statistical calculations were done using the GraphPad QuickCalcs software. Student’s *t-*test (unpaired) was used. A P value of < 0.05 was taken as significant and < 0.005 was considered extremely significant. For proteomics experiments, P values were calculated using the inbuilt statistical functions of Perseus platform.

## Additional Information

**How to cite this article:** Paul, I. *et al*. The ubiquitin ligase Cullin5^SOCS2^ regulates NDR1/STK38 stability and NF-κB transactivation. *Sci. Rep.* **7**, 42800; doi: 10.1038/srep42800 (2017).

**Publisher's note:** Springer Nature remains neutral with regard to jurisdictional claims in published maps and institutional affiliations.

## Supplementary Material

Supplementary Information

Supplementary Table S1

## Figures and Tables

**Table 1:** Upregulated proteins identified in the proteomics screen which satisfied the criteria of a fold-change of >1.5 and P-value < 0.05.

Majority protein IDs	Protein names	Gene names	P value (−Log10)	Difference (Log2)	Unique peptides	Mol. weight [kDa]
Q8K298	Actin-binding protein anillin	Anln	5.145	2.026	18	122.79
Q9CYW4	Haloacid dehalogenase-like hydrolase domain-containing protein 3	Hdhd3	1.899	1.633	4	28.027
Q3TFD2	Lysophosphatidylcholine acyltransferase 1	Lpcat1	2.301	1.605	3	54.649
Q78XF5	Oligosaccharyltransferase complex subunit OSTC	Ostc	3.778	1.604	2	16.815
Q9D3D9	ATP synthase subunit delta, mitochondrial	Atp5d	2.552	1.560	5	17.6
Q9CR41	Huntingtin-interacting protein K	Hypk	2.786	1.560	4	14.679
Q05CN2	Ribosomal RNA processing protein 1 homolog A	Rrp1	1.762	1.529	4	42.904
Q9CXG3	Peptidyl-prolyl cis-trans isomerase-like 4	Ppil4	2.440	1.485	10	57.23
Q0KL02	Triple functional domain protein	Trio	2.018	1.398	7	288.71
Q80ZX0	Protein Sec24b	Sec24b	2.144	1.358	9	135.55
Q5XF89	Probable cation-transporting ATPase 13A3	Atp13a3	2.296	1.339	3	137.47
Q9CR27	WASH complex subunit CCDC53	Ccdc53	2.265	1.323	3	21.092
F2Z4B3	Cyclin-dependent kinase 2-associated protein 1	Cdk2ap1	2.562	1.258	3	9.6308
Q4KML4	Costars family protein ABRACL	Abracl	4.853	1.254	5	9.0303
P16254	Signal recognition particle 14 kDa protein	Srp14	3.115	1.250	6	12.51
Q8CHT1	Ephexin-1	Ngef	4.329	1.243	6	71.415
Q99LC8	Translation initiation factor eIF-2B subunit alpha	Eif2b1	1.667	1.217	7	33.816
Q3TME9	Tyrosine-protein kinase Fer	Fer	3.260	1.209	4	45.071
Q6ZQK0	Condensin-2 complex subunit D3	Ncapd3	3.285	1.189	12	169.43
Q8BW84	Glutamyl-tRNA(Gln) amidotransferase subunit B, mitochondrial	Pet112	3.543	1.180	5	61.509
E9Q555	E3 ubiquitin-protein ligase RNF213	Rnf213	2.675	1.138	18	584.49
Q8R5C5	Beta-centractin	Actr1b	1.997	1.098	6	42.281
Q9DCJ5	NADH dehydrogenase [ubiquinone] 1 alpha subcomplex subunit 8	Ndufa8	3.935	1.098	5	19.992
Q8BGB7	Enolase-phosphatase E1	Enoph1	2.729	1.096	3	25.358
Q52L97	Importin subunit alpha	Kpna2	4.766	1.053	14	57.927
P62077	Mitochondrial import inner membrane translocase subunit Tim8 B	Timm8b	4.695	1.034	3	9.2864
Q99LT0	Protein dpy-30 homolog	Dpy30	3.094	1.015	4	11.213
E9PY48	Condensin-2 complex subunit H2	Ncaph2	1.970	1.010	4	65.309
Q9EPL8	Importin-7	Ipo7	4.506	0.978	20	119.49
Q9DAS3	Dynein light chain Tctex-type 1	Dynlt1f	3.252	0.960	3	11.625
Q91WK1	SPRY domain-containing protein 4	Spryd4	2.463	0.937	7	23.275
Q6P4T3	Eyes absent homolog 3	Eya3	2.519	0.924	13	57.854
Q9D1R2	Protein KTI12 homolog	Kti12	1.877	0.905	4	38.444
D5MCW4	Protein CutA	Cuta	3.344	0.897	3	16.453
Q543N6	Serine/threonine-protein phosphatase 2 A activator	Ppp2r4	2.280	0.897	15	36.71
Q9CRT8	Exportin-T	Xpot	2.742	0.888	15	109.73
A2AEB4	Mortality factor 4-like protein 2	Morf4l2	2.734	0.886	6	22.919
Q9CQK7	RWD domain-containing protein 1	Rwdd1	2.170	0.884	6	27.785
D3YYC2	Dr1-associated corepressor	Drap1	3.189	0.863	4	17.587
Q8CC06	Integrin alpha-6	Itga6	2.066	0.859	12	119.6
Q3UB06	SRSF protein kinase 1	Srpk1	4.142	0.856	10	73.088
Q9D7A8	Armadillo repeat-containing protein 1	Armc1	2.063	0.851	4	31.246
Q9CZ30	Obg-like ATPase 1	Ola1	3.529	0.846	21	44.729
P08030	Adenine phosphoribosyltransferase	Aprt	3.618	0.844	12	19.724
Q5SSZ5	Tensin-3	Tns3	3.978	0.840	1	58.725
Q3UTL4	High affinity cationic amino acid transporter 1	Slc7a1	2.364	0.835	3	67.107
Q8VDF2	E3 ubiquitin-protein ligase UHRF1	Uhrf1	3.938	0.829	20	87.555
Q9CPY1	39 S ribosomal protein L51, mitochondrial	Mrpl51	3.421	0.822	4	15.103
Q6PE15	Mycophenolic acid acyl-glucuronide esterase, mitochondrial	Abhd10	2.757	0.821	6	33.04
Q548K2	Alcohol dehydrogenase class 4 mu/sigma chain	Adh7	2.849	0.819	9	39.837
Q9CY16	28 S ribosomal protein S28, mitochondrial	Mrps28	3.494	0.817	7	20.52
G3XA18	N-acylethanolamine-hydrolyzing acid amidase	Naaa	1.903	0.807	3	39.803
Q8R5H1	Ubiquitin carboxyl-terminal hydrolase 15	Usp15	4.365	0.803	28	112.32
Q3UMQ8	H/ACA ribonucleoprotein complex non-core subunit NAF1	Naf1	2.307	0.802	3	53.245
O70591	Prefoldin subunit 2	Pfdn2	4.651	0.798	7	16.534
Q8C845	EF-hand domain-containing protein D2	Efhd2	1.873	0.795	7	26.8
Q6P9R1	ATP-dependent RNA helicase DDX51	Ddx51	3.675	0.791	14	70.367
E9PUB0	Arf-GAP with Rho-GAP domain, ANK repeat and PH domain-containing protein 1	Arap1	2.705	0.780	8	83.2
Q7TMR0	Lysosomal Pro-X carboxypeptidase	Prcp	5.910	0.777	13	55.026
Q9JJ44	DUTPase	Dut	3.584	0.777	7	17.414
Q921M8	Ubiquitin carboxyl-terminal hydrolase	Usp4	1.494	0.769	20	103.75
E9PX53	Serine/threonine-protein phosphatase 4 regulatory subunit 1	Ppp4r1	1.509	0.765	8	104.5
Q9D046	Centrin-3	Cetn3	2.665	0.762	3	19.447
Q99LZ3	DNA replication complex GINS protein SLD5	Gins4	1.888	0.760	4	25.961
Q9JKB3	Y-box-binding protein 3	Ybx3	3.254	0.757	1	38.813
Q6PDL0	Cytoplasmic dynein 1 light intermediate chain 2	Dync1li2	2.409	0.754	9	54.218
Q80 × 98	DEAH (Asp-Glu-Ala-His) box polypeptide 38	Dhx38	2.570	0.754	19	140.62
Q561N4	Ubiquitin-conjugating enzyme E2 L3	Ube2l3	1.881	0.746	1	17.861
Q8C6I2	Succinate dehydrogenase assembly factor 2, mitochondrial	Sdhaf2	3.361	0.745	6	19.431
Q8K2A7	Integrator complex subunit 10	Ints10	2.772	0.739	10	82.019
Q8K4L0	ATP-dependent RNA helicase DDX54	Ddx54	1.902	0.739	13	97.747
Q5SW19	Clustered mitochondria protein homolog	Cluh	3.374	0.736	13	148.07
Q6A009	E3 ubiquitin-protein ligase listerin	Ltn1	2.232	0.729	10	198.92
Q545F0	Macrophage migration inhibitory factor	Mif	3.086	0.728	9	12.504
Q9DBY0	Forkhead box protein P4	Foxp4	3.890	0.722	7	72.758
Q8R404	Protein QIL1	Qil1	2.812	0.722	6	13.373
Q86Y56	Dynein assembly factor 5, axonemal	Heatr2	1.698	0.722	6	93.85
Q3TBW2	39 S ribosomal protein L10, mitochondrial	Mrpl10	2.758	0.719	5	29.395
E9PWB1	Pleckstrin homology-like domain family B member 1	Phldb1	3.099	0.716	32	145.04
P70698	CTP synthase 1	Ctps1	3.766	0.715	29	66.682
Q3UMC0	Spermatogenesis-associated protein 5	Spata5	1.550	0.710	5	97.255
Q922Y1	UBX domain-containing protein 1	Ubxn1	3.403	0.709	8	33.572
Q8K4F6	Putative methyltransferase NSUN5	Nsun5	2.148	0.708	6	51.029
Q64261	Cyclin-dependent kinase 6	Cdk6	6.123	0.706	5	37.028
J3QP71	Basigin	Bsg	2.028	0.703	7	21.602
Q99M31	Heat shock 70 kDa protein 14	Hspa14	3.418	0.701	13	54.65
Q63870	Collagen alpha-1(VII) chain	Col7a1	3.142	0.699	13	295.23
A2A6Q5	Cell division cycle protein 27 homolog	Cdc27	2.122	0.696	6	91.841
Q9CRA5	Golgi phosphoprotein 3	Golph3	1.933	0.691	9	33.752
Q9JIK5	Nucleolar RNA helicase 2	Ddx21	5.098	0.690	59	93.55
Q6ZPJ3	Ubiquitin-conjugating enzyme E2 O	Ube2o	2.910	0.689	7	140.83
Q3UVH2	Tyrosine-protein kinase CSK	Csk	2.833	0.687	12	50.73
Q63829	COMM domain-containing protein 3	Commd3	2.101	0.680	5	22.037
Q9R0H0	Peroxisomal acyl-coenzyme A oxidase 1	Acox1	2.545	0.675	14	74.717
Q3UA06	Pachytene checkpoint protein 2 homolog	Trip13	1.747	0.670	8	48.376
Q8R3P6	von Willebrand factor A domain-containing protein 9	Vwa9	2.231	0.670	7	57.236
Q921Q7	Ras and Rab interactor 1	Rin1	1.329	0.664	4	83.013
P62488	DNA-directed RNA polymerase II subunit RPB7	Polr2g	2.233	0.663	7	19.294
Q6NS79	Protein diaphanous homolog 1	Diap1	2.186	0.662	16	135.84
Q8BL74	General transcription factor 3 C polypeptide 2	Gtf3c2	2.088	0.659	6	100.27
J3QMC5	Midasin	Mdn1	2.800	0.659	41	629.58
Q91VJ4	Serine/threonine-protein kinase 38	Stk38	1.602	0.659	4	54.174
Q8R126	Gltscr2 protein	Gltscr2	2.771	0.658	7	54.506
Q3U8P5	Vacuolar protein sorting-associated protein 4B	Vps4b	3.743	0.656	8	49.464
F8VQ54	Endoribonuclease Dicer	Dicer1	1.684	0.655	7	215.77
P70444	BH3-interacting domain death agonist	Bid	1.761	0.650	5	21.951
Q8C079	Striatin-interacting protein 1	Strip1	1.444	0.648	7	86.884
Q9EQ80	NIF3-like protein 1	Nif3l1	2.376	0.644	10	41.745
E9QAP7	Protein Taf	Taf4a	1.489	0.643	5	73.137
Q3THE6	Ferritin	Ftl1	3.246	0.628	2	20.695
Q3UUQ7	GPI inositol-deacylase	Pgap1	2.451	0.628	2	104.58
Q3THG5	Cyclin-dependent kinase 7	Cdk7	1.353	0.626	4	38.968
Q9D071	MMS19 nucleotide excision repair protein homolog	Mms19	1.478	0.626	8	97.458
Q9QY06	Unconventional myosin-IXb	Myo9b	3.626	0.620	11	238.83
A3KMJ8	Prefoldin subunit 3	Vbp1	3.410	0.619	9	21.963
Q6ZQH8	Nucleoporin NUP188 homolog	Nup188	2.879	0.618	21	196.69
Q9DC50	Peroxisomal carnitine O-octanoyltransferase	Crot	1.812	0.617	6	70.264
Q5PPR2	Exocyst complex component 1	Exoc1	1.615	0.616	5	100.19
Q9CZD3	Glycine–tRNA ligase	Gars	3.168	0.609	38	81.877
Q9CSZ1	BTB/POZ domain-containing protein KCTD5	Kctd5	1.377	0.606	2	18.877
Q8BVG4	Dipeptidyl peptidase 9	Dpp9	2.135	0.603	10	98
A6H5Z3	Exocyst complex component 6B	Exoc6b	1.806	0.601	5	94.128
F8VQJ3	Laminin subunit gamma-1	Lamc1	3.495	0.601	24	177.19
Q9D358	Low molecular weight phosphotyrosine protein phosphatase	Acp1	1.924	0.600	7	18.192
Q80VI1	E3 ubiquitin-protein ligase TRIM56	Trim56	2.562	0.598	10	79.512
Q3UHB1	5-nucleotidase domain-containing protein 3	Nt5dc3	2.245	0.598	11	63.17
P11440	Cyclin-dependent kinase 1	Cdk1	3.618	0.598	14	34.106
Q9JJY4	Probable ATP-dependent RNA helicase DDX20	Ddx20	1.349	0.597	8	91.709
Q99K58	Fibulin-2	Fbln2	1.933	0.596	6	126.46
Q8WTY4	Anamorsin	Ciapin1	2.119	0.596	9	33.429
Q8BJ03	Cytochrome c oxidase assembly protein COX15 homolog	Cox15	1.314	0.595	3	44.005
Q3TKX4	Probable ATP-dependent RNA helicase DDX52	Ddx52	2.903	0.595	20	67.129
Q9DB96	Neuroguidin	Ngdn	2.196	0.594	5	35.658
Q5SWU9	Acetyl-CoA carboxylase 1	Acaca	3.318	0.593	26	265.25
P46664	Adenylosuccinate synthetase isozyme 2	Adss	3.358	0.591	25	50.02
Q6WKZ8	E3 ubiquitin-protein ligase UBR2	Ubr2	2.034	0.586	18	199.2
