# Supplementary material for: The ubiquitin ligase Cullin5SOCS2 regulates NDR1/STK38 stability and NF-κB transactivation
Source: Sci Rep. 2017 Feb 20;7:42800. doi: 10.1038/srep42800 (PMC5316984; doi:10.1038/srep42800)
Supplement: Supplementary Information [file srep42800-s1.pdf]

SUPPLEMENTARY INFORMATION

The ubiquitin ligase Cullin5^{SOCS2} regulates NDR1/STK38 stability and NF-κB transactivation

Indranil Paul[1 *], Tanveer S. Batth[1], Diego Iglesias-Gato[2], Amna Al-Araimi[3], Ibrahim Al-Haddabi[3], Amira Alkharusi[3],

Gunnar Norstedt[4], Jesper V Olsen[1], Fahad Zadjali[3], Amilcar Flores-Morales[1,2 *]

SUPPLEMENTARY FIGURES

Figure S1

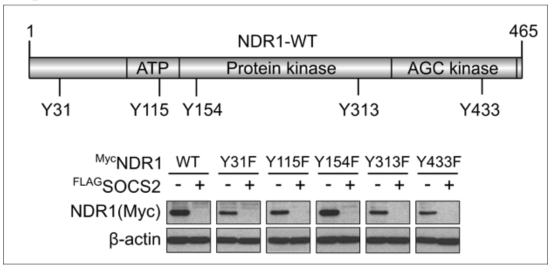

Figure S1 - Plasmids expressing Myc-NDR1 wild type and its various point mutants (Y→F, as indicated) were transfected along with FLAG-SOCS2 in HEK293T cells and lysates were probed by IB.

Figure S2

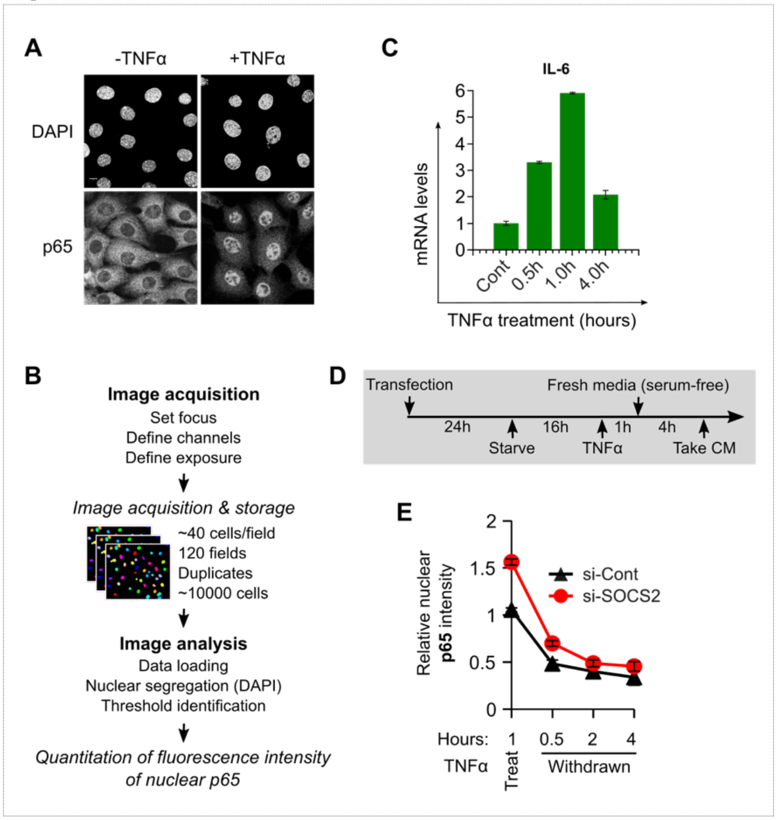

Figure S2 – (**A**) Representative confocal images for localization of p65 in MEFs treated with TNFα (10 ng/ml) for 1 hour. Magnification=1000X. (**B**) Schematic workflow for high-content quantitative imaging. (**C**) Quantitative RT–PCR analysis of NF-κB target gene IL-6 using total RNAs extracted from MEFs treated with TNFα for indicated time points. 18S rRNA was used as internal control. At 1.0 hour time point the transcriptional activity was found to be maximal. (**D**) Schematic timeline for treatments for preparation of conditioned media (CM). (**E**) MEFs were transfected with either NT siRNA or SOCS2 siRNA as shown. After 36 hours cells were pulsed with TNFα (10 ng/ml) for 1 hour. Then TNFα was withdrawn and cells were incubated in fresh serum-free media for indicated time points before being harvested and processed for HCQI.

Figure S3

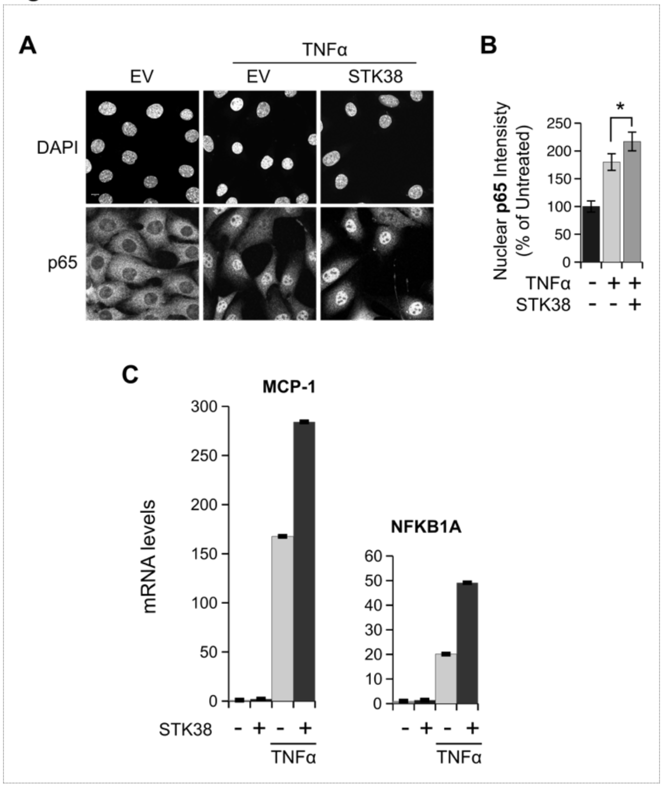

Figure S3 – (A) Representative confocal images for localization of p65 in MEFs transfected with STK38 and treated with TNFα (10 ng/ml) for 1 hour, as shown. Magnification=1000X. (**B**) Bar graph showing nuclear staining intensities of p65 from confocal images in (A) quantified using ImageJ. (**C**) Quantitative RT–PCR analysis of NF-κB target genes using total RNAs extracted from MEFs transfected with STK38 and treated with TNFα for 1 hour. 18S rRNA was used as internal control.

Figure S4

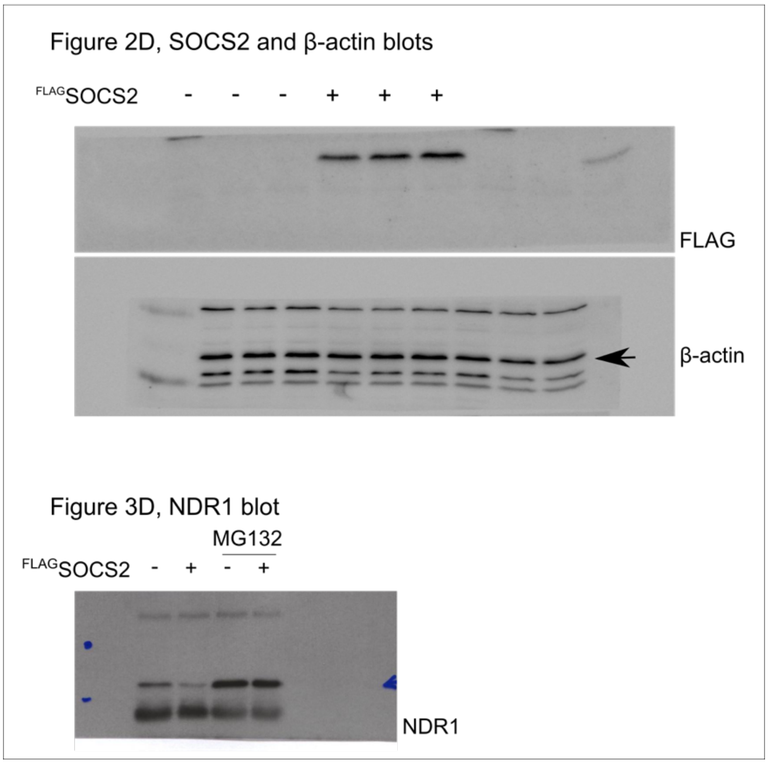

Figure S4. Uncropped blots for gels tightly cropped to show relevant bands in the main figure.

SUPPLEMENTARY TABLES

Table S1 – List of all proteins identified in the proteomic screen. Provided as a separate spreadsheet.

Table S2 - Target sequences of siRNAs used.

Name of gene	Name of siRNA	Target sequence
SOCS2	SOCS2#2	cgcattcagactacctactaa
	SOCS2#6	atgcagctatgtgaaagagaa
	SOCS2#8	atgtgtcaagtccaagcttaa
STK38	STK38#1	tgcgatatctattgaaatcaa
	STK38#3	gaggatagaatttaagactta
Non-targeting	Control	cagggtatcgacgattacaaa

Table S3 – Sequences for qPCR primers (mouse) used.

Target	Sequence (5'-3')
18S	For – gtaacccgttgaaccccatt<br>Rev - ccatccaatcggtagtagcg
STK38	For - gaccccacaagagacatacaag<br>Rev - ggctccgattctatgttccc
SOCS2	For - aggtacaggtgaacagtcccatt<br>Rev - tccagatgtgcaaggataaacg
IL-6	For - ctgatgctggtgacaaccac<br>Rev - tccacgatttcccagagaac
NFKB1A	For - accaaggctactcccctac<br>Rev - ctctcctcatcctcgctctc
MCP-1	For - cccaatgagtaggctggag<br>Rev - tctggacccattccttcttg
Arginase-1	For - ctccaagccaaagtccttagag<br>Rev - aggagctgtcattagggacatc
iNOS	For - gttctcagcccaacaatacaaga<br>Rev - gtggacgggtcgatgtcac
TNFα	For - gcctcttctcattcctgcttg<br>Rev - ctgatgagagggaggccatt

Table S4 – Histologic colitis scoring guideline.

**Feature**	**Score**	**Description**
Inflammation severity	0	None
	1	Mild
	2	Moderate
	3	Severe
Inflammation extent	0	None
	1	Mucosa
	2	Submucosa
	3	Transmural
Crypt damage	0	None
	1	Basal 1⁄3 damaged
	2	Basal 2⁄3 damaged
	3	Crypt lost
	4	Surface epithelial lost
Ulcer	4	
Lymphocyte infiltration	3	
Neutrophil infiltration	2	
Cryptitis	3	
Crypt abscess	3	
Edema	4	
Goblet cell depletion	3	
